# Skeletal muscle as a dynamic immunological niche for vaccination

**DOI:** 10.3389/fimmu.2026.1808541

**Published:** 2026-04-22

**Authors:** Moataz Noureddine, Michael Schotsaert

**Affiliations:** 1 Department of Microbiology, Icahn School of Medicine at Mount Sinai, New York, NY, United States; 2 Global Health and Emerging Pathogens Institute, Icahn School of Medicine at Mount Sinai, New York, NY, United States; 3 Graduate School of Biomedical Sciences, Icahn School of Medicine at Mount Sinai, New York, NY, United States; 4 Icahn Genomics Institute, Icahn School of Medicine at Mount Sinai, , New York, NY, United States; 5 Marc and Jennifer Lipschultz Precision Immunology Institute, Icahn School of Medicine at Mount Sinai, New York, NY, United States; 6 Department of Immunology and Immunotherapy, Icahn School of Medicine at Mount Sinai, New York, NY, United States

**Keywords:** aging, muscle biology, muscle regeneration, sarcopenia, vaccination

## Abstract

Intramuscular vaccination has long been a cornerstone of preventive medicine and has substantially reduced global mortality from infectious disease. As vaccine development has expanded to address increasingly complex targets, including chronic infections and cancer, it has become clear that vaccine efficacy depends not only on antigen design but also on the inflammatory and cellular cues present at the site of delivery. Despite this, the skeletal muscle—the most common site of vaccine administration—has received comparatively little attention as an immunological environment. Skeletal muscle is a highly dynamic, regenerative tissue whose immune composition is actively remodeled by injury, aging, and disease. How these context-dependent immune states shape vaccine uptake and downstream immune responses remains poorly understood. In this review, we examine the immune landscape of skeletal muscle in health and disease and discuss how its intrinsic regenerative programs, and their disruption in conditions such as sarcopenia, may influence intramuscular vaccine responses.

## Highlights

- Muscle is an immune-distinct landscape with potential under-explored contribution to vaccine response.
- Muscle regeneration is a well-controlled immune reaction with inflammatory cues relevant to vaccine-specific cellular and humoral responses.
- Aging alters the muscle niche, and inflammaging has the potential to change vaccine responses.
- Studying the muscle niche might highlight targets to enhance certain vaccine efficacies.

## Why discuss musculoskeletal tissue in the context vaccine development?

Despite skeletal muscle being the most common site of vaccine administration, its role in shaping vaccine-induced immune responses remains largely unexplored. Far from being immunologically inert, muscle is a dynamic tissue with tightly regulated immune programs driven by its regenerative demands and sensitivity to inflammation. Vaccination therefore occurs within an environment that both permits and constrains immune activation, potentially influencing antigen uptake, immune polarization, and response durability. As vaccines increasingly move toward personalized applications beyond infectious disease, understanding how musculoskeletal tissue contributes to vaccine efficacy becomes both timely and essential.

Furthermore, older adults are disproportionately affected by infectious diseases yet exhibit diminished responses to many intramuscular vaccines. Emerging evidence suggests that skeletal muscle is a dynamic and active immune environment whose composition is substantially altered with age. By integrating recent advances in muscle immunobiology with principles of vaccine-induced immunity, this review highlights skeletal muscle as an underexplored determinant of vaccine efficacy and a potential target for improving geriatric vaccination strategies.

## Vaccine development and current challenges

### Vaccine types

The primary goal of vaccination is to establish pre-existing immunity against an infectious agent, thereby enabling rapid recall of adaptive immune responses that would otherwise require extended periods to develop, during which the host exhibits increased morbidity or may even succumb to infection. Despite the diversity of vaccine platforms, most vaccines share a common route of administration, with skeletal muscle serving as the primary injection site.

Vaccine platforms vary depending on the infectious agent and the type of immune protection required. Live attenuated vaccines, such as the measles–mumps–rubella (MMR) vaccine, consist of weakened forms of the original pathogen and closely mimic natural infection, thereby eliciting long-lasting robust T cell– and B cell–mediated immune responses (1). However, these vaccines carry a risk of reversion to pathogenicity and are contraindicated in immunocompromised individuals which restricts their use for some pathogens (2).

Inactivated vaccines utilize whole pathogens that have been rendered non-infectious through heat, radiation, or chemical treatment, providing broad antigenic exposure capable of inducing adaptive immune responses (2). Subunit vaccines, by contrast, contain selected immunogenic components of a pathogen and primarily induce neutralizing antibody responses while offering improved safety profiles (2).

More recently, the SARS-CoV-2 pandemic accelerated the development and global deployment of mRNA lipid nanoparticle vaccines. These platforms enabled rapid, large-scale vaccine production in response to a novel pathogen and significantly reduced COVID-19–associated morbidity and mortality worldwide (3).

Together with emerging technologies such as next-generation DNA-based vaccines (2), these diverse vaccine platforms have established vaccination as a cornerstone of modern public health. Continued innovation in vaccine design and delivery holds considerable promise for addressing longstanding challenges, including HIV, cancer, and autoimmune diseases.

### Adjuvants and self-adjuvating vaccines

Despite significant advances in vaccine technology, several limitations continue to constrain the efficacy of traditional vaccine platforms, underscoring lessons that can be drawn from newer formulations such as mRNA lipid nanoparticle vaccines. A major challenge in vaccine development is the insufficient induction of inflammatory responses required to enhance the immunogenicity of vaccine foreign antigens. This limitation is particularly pronounced for inactivated and subunit vaccines, which comprise a substantial proportion of vaccines currently in clinical use.

Insufficient inflammation at the site of vaccination, particularly within muscle tissue, has been associated with impaired antigen uptake and transport by antigen-presenting cells (APCs) to draining lymph nodes, resulting in suboptimal humoral and cellular immune responses. To overcome this limitation, a limited set of adjuvants has been employed to enhance vaccine immunogenicity. Clinically approved adjuvants primarily include aluminum salts, emulsions, particulate formulations, and Toll-like receptor (TLR) agonists, with some formulations combining multiple adjuvants to achieve optimal efficacy (4).

Aluminum salts (alum) were first approved for clinical use in the 1920s in combination with the diphtheria vaccine (4). Although widely used, the precise mechanisms underlying alum’s adjuvant activity remain incompletely understood and are thought to involve multiple processes. Alum has been shown to promote adsorption of antigens, facilitating phagocytosis by APCs and enabling the formation of a prolonged antigen depot (5, 6). In addition, alum administration has been associated with localized muscle fiber necrosis and recruitment of macrophages, neutrophils, and eosinophils (5, 6). Macrophages responding to alum crystals may differentiate toward dendritic cell–like phenotypes, thereby enhancing antigen uptake and priming within the draining lymphatic system (5, 6). Furthermore, Alum has been shown to activate intracellular Napl3 inflammasome in its immunostimulatory mechanism (7).

Emulsion adjuvants consist of water-in-oil or oil-in-water mixtures stabilized by surfactants (5). MF59 is an oil-in-water emulsion adjuvant approved for use in trivalent and quadrivalent seasonal influenza vaccines, particularly in older adults to counteract age-associated declines in vaccine efficacy. MF59 is also under investigation in combination with other vaccine platforms, including the spike glycoprotein clamp vaccine for SARS-CoV-2 (8). Although the precise mechanism of action of MF59 remains unclear, it is well established that MF59 induces a robust inflammatory response at the injection site, characterized by the recruitment of macrophages, neutrophils, eosinophils, and lymphocytes (9, 10). These infiltrating immune cells are largely responsible for enhanced antigen uptake and subsequent priming in draining lymph nodes (9). MF59 has also been shown to induce ATP release from muscle tissue, consistent with localized cellular stress or necrosis (11). However, the upstream signals driving MF59-induced inflammation remain incompletely defined.

In contrast, CpG oligodeoxynucleotides and other TLR agonists exhibit more clearly defined mechanisms of action. These adjuvants directly activate TLRs, triggering downstream signaling pathways mediated by Myeloid differentiation primary response 88 (MyD88) and/or TIR-domain-containing adaptor-inducing interferon-β (TRIF) (12). This signaling results in the induction of type I interferons, such as IFNα and IFNβ, and pro-inflammatory cytokines including IL-1β and IL-12 (12). These cytokines enhance antigen trafficking to lymphoid tissues and promote robust T cell–mediated immune responses to vaccine antigens (12). Despite their well-characterized mechanisms, relatively few CpG and TLR agonists have been approved for clinical use. Currently, only TLR4 agonists, such as monophosphoryl lipid A (MPLA), are licensed for use in vaccines against malaria and herpes zoster (12).

### T-helper polarization

An additional challenge in vaccine development is the induction of the appropriate type of immunity against the target pathogen. Immune response profiles are largely determined by the polarization of CD4^+^ T helper cells toward Th1, Th2, or Th17 lineages. Each of these response types is evolutionarily adapted to distinct classes of pathogens, exploiting specific pathogen vulnerabilities to promote effective immune clearance.

A Th1 response is characterized by antigen-specific T-bet^+^ CD4^+^ T helper cells (Th1 cells) (13). Th1 cells produce high levels of IFNγ, which promotes the expansion and cytotoxic function of antigen specific CD8^+^ T cells (Tc1 cells). Differentiation of naïve CD4^+^ T cells into Th1 cells occurs primarily under the influence of IL-12 and IFNγ following dendritic cell–mediated major histocompatibility complex (MHC) class II antigen presentation (14). The dendritic cells responsible for priming Th1 responses are predominantly conventional type 1 dendritic cells (cDC1s), although monocyte-derived dendritic cells (moDCs) can also contribute (14). Recent work has further demonstrated that conventional type 2 dendritic cells (cDC2s) can acquire cDC1-like features and promote Th1 polarization under specific inflammatory conditions (14).

Th1 priming occurs within secondary lymphoid organs, such as draining lymph nodes, necessitating prior recruitment or differentiation of cDC1s at the site of infection before migration to lymphoid tissues. The signals governing cDC1 recruitment or differentiation in peripheral tissues remain an active area of investigation. In cancer, cDC1 recruitment has been shown to depend on CCL7 (15). Whereas during influenza infection of the lung, cDC1s differentiate from pre-dendritic cells in response to CCL2 and type I interferons (16–18).

Functionally, Th1 immunity promotes antigen-specific cytotoxic clearance of infected or malignant cells. This is mediated either directly by cytotoxic CD8^+^ T cells or indirectly through antibody-dependent cellular cytotoxicity (ADCC) mediated by natural killer (NK) cells (17). Th1 responses are therefore particularly effective against intracellular pathogens such as viruses and represent the dominant immune response elicited during natural respiratory viral infections (19).

In contrast, a Th2 response is characterized by antigen-specific GATA3^+^ CD4^+^ T helper cells (Th2 cells) (13). Th2 cells produce IL-4, IL-5, and IL-13, cytokines that promote tissue repair, anti-parasitic immunity, and, in certain contexts, allergic inflammation (13). The mechanisms driving Th2 differentiation remain incompletely understood but involve multiple myeloid and granulocyte populations as well as distinct inflammatory cues. For example, fibroblast-derived IL-33 in peripheral tissues contributes to tissue repair and Th2 polarization (20). In addition, IL-4 produced by basophils and group 2 innate lymphoid cells (ILC2s) has been implicated in promoting Th2 differentiation (20).

Th2 responses are essential for efficient wound repair and protection against helminth infections but can become pathogenic in the context of hypersensitivity and IgE-mediated diseases, like atopic asthma where antigen-dependent mast cell degranulation leads to histamine-dependent bronchial constriction (21, 22). In infectious settings, Th2 responses are generally ineffective against intracellular pathogens; however, they can promote the generation of neutralizing IgG antibodies with limited ADCC capacity, which may block viral entry and prevent infection at early stages (23).

A Th17 response primarily functions to maintain barrier and mucosal immunity against bacterial and fungal pathogens. Th17 cells are defined by expression of the transcription factor RORγt and differentiate from naïve CD4^+^ T cells in response to IL-6, TGF-β, IL-21, and IL-23 (24). Th17 differentiation is inhibited by cytokines associated with Th1 and Th2 responses, such as IL-2 and IL-4 (24). When dysregulated, Th17 responses play a critical role in autoimmune diseases, including type 1 diabetes, systemic lupus erythematosus, and asthma (24). Th17 immunity promotes neutrophil recruitment and inflammatory amplification at sites of infection and therefore requires tight regulation and balance with regulatory T cell (Treg) responses to prevent immunopathology (25).

Although substantial progress has been made in promoting Th1-biased immunity—particularly through lipid nanoparticle–based vaccine platforms—a significant proportion of vaccine formulations, including subunit and inactivated vaccines, fail to elicit robust Th1 responses, and instead promote a Th2 response even when they are targeting viral pathogens (26). Even though the response is not what is observed after natural respiratory viral infection, Th2-biased responses can nevertheless confer protection in certain contexts, including influenza infection, largely through the generation of neutralizing antibodies (27).

Importantly, vaccine efficacy is enhanced when T cell responses are selectively programmed to match the immunological requirements of the target pathogen. For example, Th1 cell response allows clearing intracellular viral particles, and shows increased cross-reactivity compared to antibodies, meaning that it is able to infer protection against a larger repertoire of viral strains in case of antigenic drift (28). Furthermore, in some rare cases inducing the incorrect T-cell response can enhance disease or render the vaccine inefficient. For example, vaccine-induced Th2 responses have been associated with an increased risk of vaccine-associated enhanced respiratory disease (VAERD) in a preclinical model for COVID-19 (29). Th2 responses to vaccines have also been an obstacle in expanding vaccine platforms to other diseases like cancer, where what is needed is a Th1 response capable of clearing mutated cells (30). There is currently no clear mechanism connecting initial immune responses at the injection site to vaccine-specific T-helper response differentiation. This challenge could be addressed by investigating the intrinsic immunological criteria of the muscle environment where the vast majority of vaccines are delivered, and how such an environment potentially contributes to T-cell response.

### Aging and vaccine efficacy

Aging also presents additional challenges, as vaccine efficacy declines, rendering vaccines less protective in older individuals than in younger populations. This is important because with age, vaccination becomes increasingly important for preventing severe disease caused by pathogens that exhibit age-associated increases in pathogenicity, such as seasonal influenza. For example, individuals aged ≥65 years account for approximately half of all reported influenza-associated hospitalizations, despite representing the age group with the highest rates of seasonal influenza vaccination (31, 32).

In the aging population, multiple noteworthy systemic alterations occur that may impair vaccine-induced immune responses. Lymph nodes decline in both number and size and become increasingly disorganized with age, limiting their capacity to support germinal center formation of comparable size and efficiency to those observed in younger individuals (33). Bone marrow becomes progressively more adipose with age (34) and exhibits alterations in the hematopoietic stem cell niche that favor myelopoiesis at the expense of lymphopoiesis (35). As a result, the lymphocyte compartment is skewed toward memory and effector T cells, with a reduced pool of naïve T cells available to mount *de novo* adaptive immune responses against novel pathogens (36).

In the context of inactivated influenza vaccination, humoral immune responses are diminished and seroconversion occurs more slowly in older individuals compared with younger populations (31). However, this trend is not universal across vaccine platforms. For example, antibody responses to lipid nanoparticle–based SARS-CoV-2 vaccines have been reported to be enhanced in older individuals relative to younger cohorts (37). The mechanisms underlying these differential effects of aging on humoral immunity across vaccine types remain poorly understood. Aging has also been shown to influence CD4^+^ T helper cell polarization, with a relative skewing toward Th1 phenotypes and a concomitant reduction in Th2 responses (38, 39). Whether the qualitative nature of vaccine-induced T helper responses contributes to age-associated declines in vaccine efficacy remains unclear.

Evaluating vaccine efficacy in older populations is further complicated by the heterogeneous effects of aging across organs and tissues, which can alter local immune responses to infection. For example, lung epithelial cells in aged individuals exhibit a reduced capacity to support viral antigen presentation and T cell function (40). In addition, immune responses in the aged lung are delayed and less effective at clearing infections (41). Consequently, the development of effective geriatric vaccines is complex, requiring not only enhancement of systemic cellular and humoral immune responses but also the promotion of tissue-specific inflammatory environments capable of overcoming age-associated organ dysfunction. One organ that is still underexplored in the context of aging and vaccine response is musculoskeletal tissue as an injection site. Intramuscular changes in immune response to different adjuvants like MF59 is underexplored, and little associations are made between age-associated changes with vaccine efficacy and initial intramuscular immune responses.

## The immune landscape of healthy skeletal muscle

In order to understand the potential contributions of the muscle environment to intramuscular vaccine responses, we first describe the muscle immune niche at baseline and during regeneration at health. This allows a better understanding of muscle-specific immune networks that could occur after vaccination. Since it is a factor in muscle health, we then describe how the muscle environment changes with age at baseline and during regeneration. This allows a better understanding of aging muscle-specific immune networks that could occur after geriatric vaccination.

*Baseline*.

**Figure 1:**
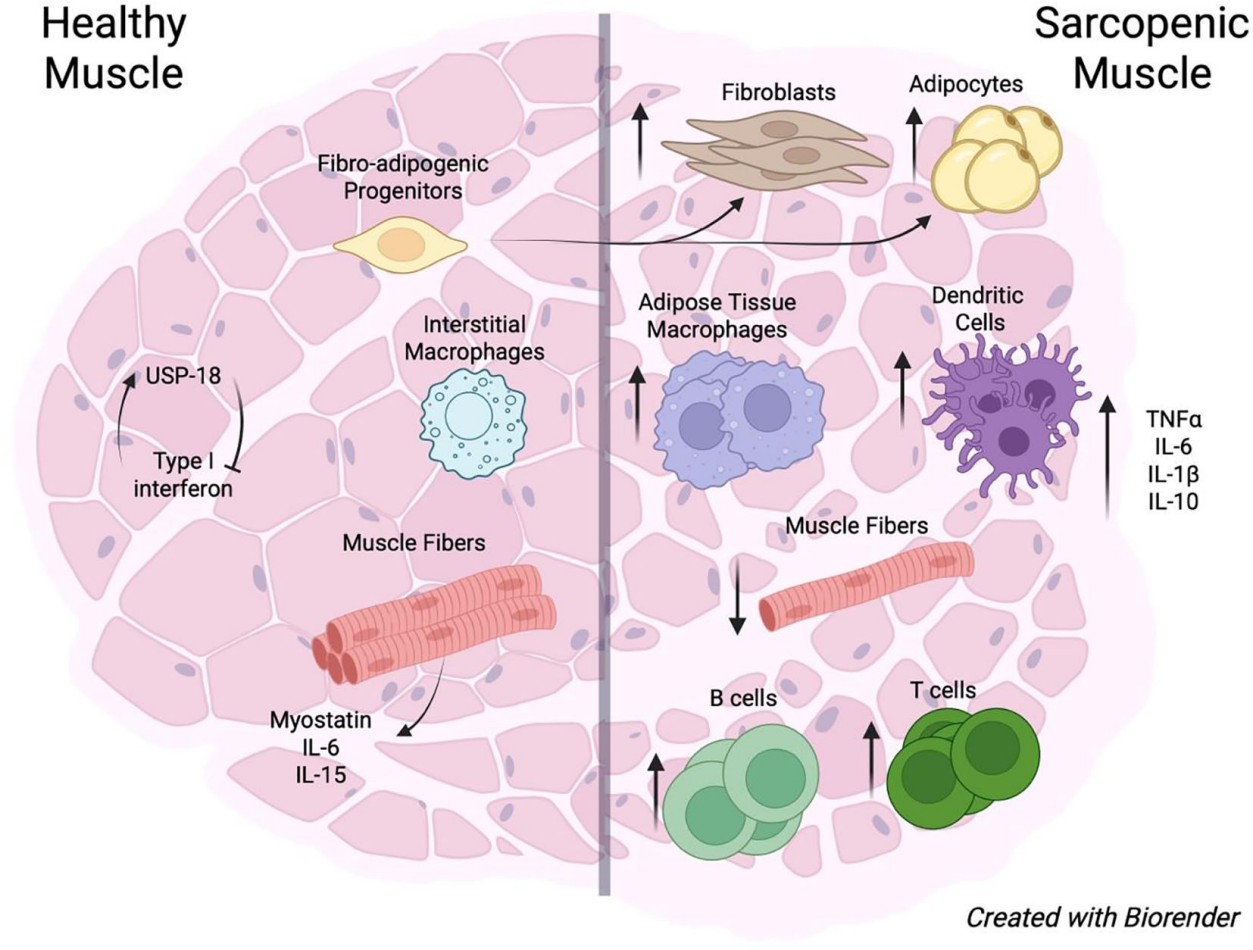
Healthy and sarcopenic muscle immune landscapes. In healthy skeletal muscle, type I interferon signaling is tightly regulated, in part through expression of the negative regulator USP18; whether this regulatory mechanism is altered with aging remains unknown. As muscle progresses toward sarcopenia, fibro-adipogenic progenitors (FAPs) increasingly differentiate into fibroblasts and adipocytes, leading to expansion of the stromal and adipogenic compartments. Concurrently, interstitial macrophages which are the predominant resident macrophage population in healthy muscle are progressively replaced by adipose tissue–associated macrophages that accumulate in aged muscle. In addition, B cells, T cells, and dendritic cells increase in abundance, contributing to a chronic low-grade inflammatory state commonly referred to as inflammaging. Aging is also associated with reduced muscle fiber size and density, accompanied by altered production of myokines, including myostatin, IL-6, and IL-15. Collectively, these changes give rise to a sarcopenic muscle microenvironment characterized by elevated baseline levels of TNF-α, IL-6, IL-1β, and IL-10 relative to healthy muscle. (Created in BioRender. Laghlali, G. (2026) https://BioRender.com/ayoultl) .

At the homeostatic baseline, skeletal muscle consists predominantly of myofibers organized into bundles (fascicles) that are separated by connective tissue layers (42) (**Figure 1**). Muscle fibers are maintained by a stem cell niche composed of satellite cells, which proliferate and differentiate into myoblasts that subsequently fuse to form multinucleated myotubes and mature muscle fibers (43). Under homeostatic conditions, satellite cells are largely quiescent and reside in close association with muscle fibers.

In addition to muscle fibers, skeletal muscle is highly vascularized. Larger venules are located within the epimysium which is the connective tissue sheath surrounding the entire muscle while smaller venules are found between fascicles, and capillaries course between individual muscle fibers (42). Consequently, muscle tissue contains a substantial population of endothelial cells (44). At baseline, immune cell presence is limited and consists predominantly of interstitial macrophages located near venules, as well as resident macrophages within the surrounding connective tissue (epimysium and perimysium), and thus not necessarily in close proximity to muscle fibers (45, 46).

Skeletal muscle is also innervated by the nervous system, including motor, sympathetic, and sensory nerve fibers, with neuromuscular junctions formed at the level of individual muscle fibers (47–49). Finally, muscle tissue contains fibro-adipogenic progenitors (FAPs), a mesenchymal progenitor population capable of differentiating into fibroblasts, adipocytes, and osteogenic cells (50).

### Injury and regeneration

**Figure 2:**
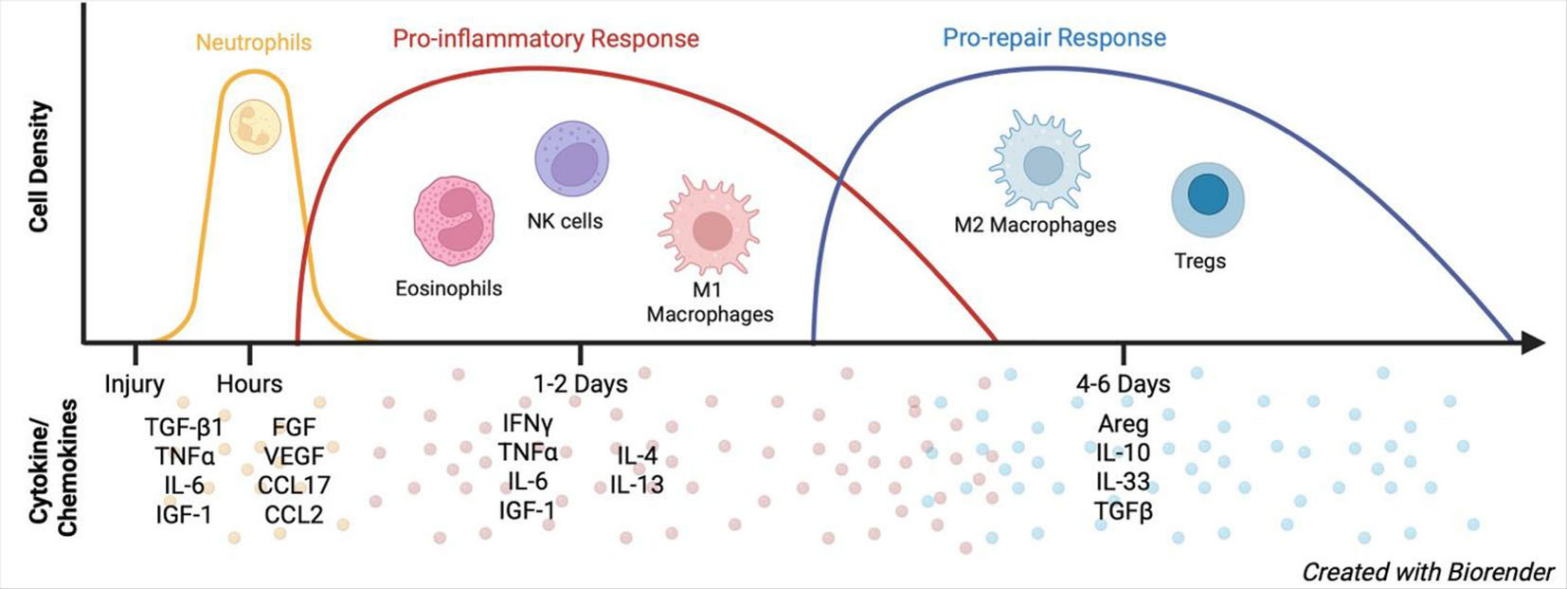
Immune landscape of healthy muscle regeneration. In healthy skeletal muscle, neutrophils infiltrate the tissue within hours following injury and express TGF-β1, TNFα, IL-6, IGF-1, FGF, VEGF, CCL7, and CCL2. This early neutrophil-driven environment initiates a pro-inflammatory response characterized by the accumulation of eosinophils, NK cells, and classically activated (M1) macrophages. The pro-inflammatory phase is associated with increased levels of IFNγ, TNFα, IL-6, IGF-1, IL-4, and IL-13 and peaks within 1–2 days after injury. This phase is followed by a pro-repair response marked by regulatory T cell (Treg) infiltration and repolarization of the macrophage niche toward anti-inflammatory (M2) phenotype. The pro-repair phase is characterized by elevated expression of amphiregulin (Areg), IL-10, IL-33, and TGF-β and peaks between 4- and 6-days post-injury. As regeneration resolves, immune cell presence in the muscle declines, returning to pre-injury baseline levels. (Created in BioRender. Laghlali, G. (2026) https://BioRender.com/vsfcsla) .

Murine models of muscle injury commonly rely on mechanical insults, such as crush injury, or chemical insults, including injections of BaCl_2_, glycerol, or cardiotoxin (CTX) (51–53). Across these models, a robust regenerative program has been described that is highly immune dependent and, more specifically, macrophage led (54) (**Figure 2**). Muscle regeneration proceeds through a biphasic response, beginning with a pro-inflammatory phase and transitioning to a pro-repair phase (55).

The initial pro-inflammatory response is characterized by infiltration of NK cells, CD8^+^ T cells, neutrophils, eosinophils, and pro-inflammatory monocyte-derived macrophages (M1) (56). This phase is initiated within hours after injury and can persist for several days depending on the injury model, after which it is followed by a pro-repair phase marked by regulatory T cell (Treg) infiltration and repolarization of the macrophage niche toward an anti-inflammatory (M2) phenotype (57, 58).

Each immune cell type plays a distinct and essential role in this regenerative cascade. Neutrophils are the first immune cells to infiltrate injured muscle, appearing within hours after CTX-mediated injury via platelet-derived CXCL7 signaling (59). Upon infiltration, neutrophils express growth factors such as fibroblast growth factor (FGF), insulin-like growth factor-1 (IGF-1), vascular endothelial growth factor (VEGF), and transforming growth factor-β1 (TGF-β1), as well as pro-inflammatory cytokines including tumor necrosis factor alpha (TNFα) and IL-6 (60). Neutrophils also produce chemokines such as CCL17 and CCL2, the latter being critical for monocyte recruitment and initiation of the macrophage response to muscle injury (60).

Recruited monocytes subsequently infiltrate the muscle and polarize toward an M1 macrophage phenotype under the influence of NK cell–derived IFNγ and TNFα (61). M1 macrophages play a crucial role in debris clearance following injury (62) and reinforce the pro-inflammatory milieu through continued expression of IFNγ (63). In addition, M1 macrophages secrete IL-6, which promotes myoblast proliferation (64), and IGF-1, which stimulates the expansion of myogenic precursor cells (65).

Concurrently, eosinophils accumulate within injured muscle and produce IL-4 and IL-13, which act on fibro-adipogenic progenitors (FAPs) to promote a phagocytic phenotype and limit fatty degeneration during regeneration (66). Eosinophil-derived IL-4 further induces IL-33 expression in FAPs (66).

IL-33 production by FAPs initiates the pro-regenerative phase by recruiting ST2^+^ Tregs (67, 68). Recruited Tregs subsequently drive macrophage niche repolarization from an M1 toward a reparative M2 phenotype through multiple mechanisms, including secretion of IL-10 and amphiregulin (Areg), as well as suppression of IFNγ expression (68, 69). Tregs additionally support myoblast survival and proliferation through Areg-mediated signaling (68). Newly polarized M2 macrophages then produce IL-10 and TGF-β, thereby promoting muscle fiber growth and regeneration (70). As regeneration resolves, immune cell numbers gradually decline, restoring the immune-scarce environment characteristic of uninjured muscle.

### The immune landscape of aging skeletal muscle

With age, muscle regenerative capacity declines, ultimately contributing to the development of sarcopenia (71, 72). Sarcopenia is associated with intrinsic alterations in musculoskeletal tissues, including mitochondrial dysfunction and stem cell senescence (73, 74), as well as with changes at the muscle–immune interface that promote chronic inflammation (75) (**Figure 1**).

At baseline, aging induces widespread changes across most cellular components of the muscle niche. Muscle fibers decline both in number (76), and functional quality (74, 77). a phenomenon that has been partly attributed to age-associated transcriptional alterations affecting muscle fiber differentiation and metabolism (78). In addition, aging skeletal muscle exhibits increased fatty infiltration and fibrosis, driven by the expansion of adipocytes and fibroblasts within the niche (79). These structural changes have been linked to age-associated dysregulation of fibro-adipogenic progenitors (FAPs), which increasingly differentiate into fibroblasts and adipocytes during muscle regeneration (80, 81).

Adipose tissue plays a central role in muscle inflammaging and the progression of sarcopenia (82). Expansion of adipose tissue within skeletal muscle creates a permissive niche for intermuscular adipose tissue macrophages (IMATMs) (83). Myokines, including MCP-1, myostatin, TNFα, IL-10, and IL-1β, act on IMATMs to stimulate the production of pro-inflammatory mediators such as TNFα, IL-1β, IL-6, and monocyte chemoattractant protein (84). This positive feedback loop exacerbates muscle fibrosis and directly contributes to muscle inflammaging (84). In addition to IMATMs, pro-inflammatory macrophages are expanded in aged skeletal muscle tissue at baseline (85).

Beyond macrophages, muscle inflammaging is characterized by increased baseline abundance of other immune cell populations, including dendritic cells (e.g., monocyte-derived DCs and cDC1s), lymphocytes (e.g., B and T cells), and neutrophils (75). Collectively, these alterations distinguish aged skeletal muscle from the immunologically quiescent state observed in healthy young muscle.

Upon injury and with age, the immune response to muscle injury changes markedly and becomes less effective at supporting muscle fiber regeneration (75). These age-associated alterations are evident during both the early pro-inflammatory and the later anti-inflammatory phases of regeneration (75). At baseline, neutrophils are enriched in aged muscle; however, following injury, their accumulation during the early pro-inflammatory phase is reduced in aged mice compared with young mice (75). Similarly, eosinophil numbers are diminished during both the pro-inflammatory and anti-inflammatory stages of regeneration in aged muscle (75). Reduced eosinophil presence during muscle regeneration promotes fibro-adipogenic progenitor (FAP) differentiation toward adipocytes, leading to increased fatty infiltration in aged muscle following injury (66, 86).

Altered immune cell accumulation during the early pro-inflammatory phase of muscle regeneration is accompanied by changes in cytokine expression and macrophage phenotypes. For example, IL-4 levels are reduced during the early inflammatory stage as a consequence of decreased eosinophil infiltration (86), whereas pro-inflammatory cytokines such as IFNγ and TNFα are increased (87). These cytokine changes are associated with a shift in the macrophage niche, characterized by an increased presence of pro-fibrotic macrophages at this stage (87). Notably, the density of pro-inflammatory macrophages appears to be largely unaffected by aging during this phase (87).

Efficient muscle regeneration requires a timely transition from a pro-inflammatory environment to a pro-repair or anti-inflammatory milieu; however, this transition is delayed or impaired in aged muscle (67, 68). This process is predominantly regulated within the macrophage niche and depends on IL-33–mediated recruitment of Tregs (54, 67). With age, FAPs exhibit reduced IL-33 expression, resulting in diminished Treg recruitment and impaired macrophage repolarization toward a pro-repair or anti-inflammatory phenotype (88). Consequently, IL-33 has emerged as a target of interest, with studies demonstrating beneficial effects of IL-33 supplementation in atrophic or regenerating aged skeletal muscle (88–90). Failure to establish an anti-inflammatory macrophage niche leads to reduced expression of MANF and selenoprotein P, thereby impairing efficient muscle fiber regeneration (91, 92). Persistence of a pro-inflammatory macrophage phenotype further contributes to sustained expression of cytokines such as IFNγ and TNFα, rather than pro-repair cytokines like IL-4, during the regenerative response (69).

## Muscle biology–driven perspectives on vaccine responses

### Healthy muscle

The immunological composition of skeletal muscle at homeostatic baseline is distinct and highly relevant for intramuscular vaccination. Under steady-state conditions, type I interferon signaling in myotubes is tightly regulated through expression of the interferon inhibitor Ubiquitin-specific peptidase 18 (USP18) (93, 94), which prevents the detrimental effects of sustained type I interferon signaling on myotube formation and muscle maintenance (95). This myotube-intrinsic regulatory program has not been extensively investigated in the context of vaccine development, including whether it contributes to the limited ability of intramuscular administration of soluble protein vaccines to induce robust CD8^+^ T cell responses (96). In contrast, lipid nanoparticle (LNP)–based vaccines have been shown to induce IFNβ expression in muscle fibroblasts, a response that correlates with vaccine-specific Th1 cellular immunity (97). These findings highlight muscle fibroblasts as a potential cellular target for future vaccine formulations designed to elicit Th1-biased cellular immune responses, such as cancer vaccines.

In addition to its cellular composition, skeletal muscle maintains low but detectable baseline levels of cytokines and myokines that support motor function and are further induced by exercise independently of tissue injury (98). These myokines include myostatin, irisin, IL-6, brain-derived neurotrophic factor (BDNF), IL-15, myonectin (CTRP15), decorin, fibroblast growth factor 21 (FGF21), and secreted protein acidic and rich in cysteine (SPARC) (98). Notably, many of these factors exert immunomodulatory effects on diverse immune cell populations. IL-6, for example, is essential for muscle homeostasis and is robustly secreted systemically by myotubes in response to muscle contraction (99, 100). Beyond its metabolic roles, IL-6 significantly influences T cell immunity by downregulating IFNγ expression through SOCS1 induction and enhancing IL-4 production via Nuclear Factor of Activated T-cells (NFAT)-dependent pathways in CD4^+^ T helper cells (101). Myostatin has likewise been implicated in promoting inflammatory responses in experimental arthritis through effects on fibro-adipogenic progenitors (102). In contrast, IL-15 supports CD8^+^ T cell survival and may contribute to the maintenance of Th1-biased immune responses (103, 104).

Despite these observations, the immunological programs governing skeletal muscle at steady state remain incompletely understood and largely unexploited in vaccine and adjuvant design. For instance, increased circulating IL-15 levels following vaccination have been correlated with enhanced vaccine efficacy; however, it remains unclear whether IL-15 is produced locally within muscle tissue and whether its expression can be therapeutically modulated at the injection site to improve vaccine responses (105). Similarly, IL-6 levels increase at the muscle injection site following administration of lipid nanoparticle–based vaccines, yet the mechanisms driving IL-6 induction and its precise role in shaping vaccine-induced immunity remain controversial (106). Furthermore, exercise after vaccination has been shown to boost humoral response to vaccination, but the potential role of myokines and the muscle environment remains underexplored (107, 108).

Upon injury, muscle regeneration is initiated immediately following injury and can persist for seven days or longer, depending on the mode and severity of injury. During the early regenerative phase, pro-inflammatory monocyte-derived macrophages and dendritic cells infiltrate the muscle, increasing the local abundance of antigen-presenting cells (APCs). Whether muscle injury–induced APC recruitment enhances vaccine antigen uptake and trafficking to draining lymph nodes has not been systematically investigated (75). At this stage, the regenerative milieu is strongly pro-inflammatory and enriched in cytokines such as IFNγ, TNFα, IL-4, and IL-13, which have the potential to influence T helper cell polarization independently of their concurrent presence within draining lymph nodes.

For example, dendritic cell–derived IFNγ has been implicated in promoting Th1 differentiation in response to foreign antigens; however, exposure of dendritic cells to IFNγ at peripheral inflammatory sites has also been shown to favor regulatory T cell (Treg) induction and suppress Th1 responses (109–111). Conversely, IL-4 and IL-13 are classically associated with skewing immune responses toward a Th2 phenotype and promoting IgE antibody production when present within lymph nodes during antigen priming (112, 113). IL-4 can additionally promote type 2 immunity at sites of inflammation by enhancing cDC2 survival (114), while IL-13 has been shown in the skin to directly drive cDC2-dependent Th2 polarization (115).

Within several days after injury, the regenerative response transitions toward an anti-inflammatory milieu characterized by elevated IL-10 and TGF-β expression, largely produced by infiltrating Tregs (68). When IL-10 and TGF-β are present during antigen priming in draining lymph nodes, they can suppress effector T cell differentiation and promote Treg induction. Recent studies have demonstrated that muscle injury increases a population of muscle-associated Tregs in muscle-draining lymph nodes; however, the implications of this response for vaccination outcomes remain unexplored (68).

As a result, the effects of inducing muscle injury at the time of vaccination on immune responses and vaccine efficacy remain unknown. Given the dynamic and phase-specific cytokine environments present during muscle regeneration, it is challenging to predict whether concomitant muscle injury would preferentially promote cellular, humoral, regulatory, or mixed vaccine-induced immune responses.

Nevertheless, insights from adjuvant research suggest potential links between muscle immune kinetics and vaccine response polarization. Notably, adjuvant-induced muscle necrosis and release of damage-associated molecular patterns (DAMPs) have been observed following administration of several clinically approved adjuvants, including alum and MF59 (5, 11). Moreover, immune cell recruitment kinetics resembling those of muscle regeneration have been reported following MF59 administration, which is used in quadrivalent inactivated seasonal influenza vaccines (QIV) in individuals aged 65 years and older. MF59 injection induces rapid neutrophil infiltration within hours, followed by increased monocyte and eosinophil accumulation within one day, closely mirroring regenerative muscle responses (9, 10). MF59 administration is also associated with elevated IL-4 and IL-10 levels at the injection site, cytokines commonly observed during muscle injury and regeneration (116).

MF59 enhances vaccine-specific antibody titers and has been shown in some models to bias immune responses toward a Th2 phenotype (117), However, its precise mechanism of action remains incompletely understood. In particular, it is unclear whether MF59 directly induces muscle injury and whether engagement of muscle regenerative pathways contributes to its ability to modulate B cell and T cell responses.

### Aging muscle

Humoral and cellular immune responses to vaccination are known to change with age. Antibody and T cell responses to multiple vaccine formulations, including seasonal influenza vaccines, decline in older individuals compared with younger populations (118, 119). This decline is associated with increased rates of influenza infection and poorer clinical outcomes, including hospitalization and mortality (118, 119). Numerous factors have been investigated as contributors to age-associated reductions in vaccine responsiveness.

Despite these insights, it remains unclear whether the increasingly inflammatory environment of aged skeletal muscle influences vaccine-induced immune responses or whether the accumulation of immune cells within muscle tissue with age contributes to altered vaccination outcomes. Dendritic cells, for example, are central drivers of vaccine-induced immunity (120), yet age-associated changes in dendritic cell abundance or function within skeletal muscle have not been examined in the context of intramuscular vaccination. It is therefore unknown whether these cells respond to vaccine administration in aged muscle or whether they could be targeted to enhance immune responses in geriatric vaccine formulations. Similarly, although adipose tissue expansion in obesity has been shown to impair vaccine-specific B cell responses (121), the impact of age-associated accumulation of intramuscular adipose tissue on vaccination efficacy has not yet been investigated.

Although aged skeletal muscle exhibits increased immune cell presence at baseline, age-associated alterations in muscle regeneration are characterized by reduced recruitment of dendritic cells, regeneration-associated macrophages, eosinophils, and neutrophils following injury (75). These changes are associated with an impaired regenerative response that favors fibrosis over effective myotube regeneration. It remains unclear whether these defects are specific to muscle injury or instead reflect broader age-related impairments in immune cell recruitment pathways.

In addition, recruitment of Tregs to injured muscle is diminished with age, limiting the transition from a pro-inflammatory to a pro-repair milieu that is required for effective regeneration (67, 68). Consequently, the aged muscle microenvironment is enriched in pro-inflammatory cytokines such as IFNγ and TNFα, while being deficient in pro-regenerative signals including IL-4, IL-13, and IL-33 (67, 75). Any investigation into the effects of inducing muscle injury in conjunction with vaccination must therefore account for age, as cytokines that shape both cellular and humoral immune responses are altered in aged muscle. In this context, transiently rejuvenating muscle immune responses during vaccination may represent a promising strategy to enhance vaccine efficacy in older individuals.

Understanding the role of muscle regenerative pathways is also of direct clinical relevance. MF59 is currently used as an adjuvant in seasonal inactivated influenza vaccines for older adults; however, its inflammatory mechanisms have largely been characterized in young mouse models (9, 10). It remains unknown whether MF59-induced immune responses are altered with age and whether such changes influence its adjuvant efficacy. Moreover, the potential consequences of adjuvant-mediated muscle injury in older individuals, particularly in the context of sarcopenia, are poorly understood and warrant further investigation.

## Concluding remarks

As vaccines become increasingly personalized and expand beyond infectious diseases to include applications such as cancer and autoimmune disorders, it is increasingly evident that the musculoskeletal injection site represents an underexplored determinant of vaccine efficacy and a promising target for modulating both the magnitude and quality of vaccine-induced immunity.

Skeletal muscle presents a dynamic and unique immune landscape that is tightly linked to its regenerative capacity. Under homeostatic conditions, muscle exhibits low immune cell abundance, yet following injury it can rapidly transition to an immune-rich environment dominated by coordinated inflammatory and reparative responses. Given the essential motor function of muscle, injury and repair are frequent physiological events, and the associated immune programs are finely tuned to support efficient regeneration. Vaccination therefore occurs within a tissue environment in which specific immune pathways are preferentially engaged, while others may be actively constrained. Despite this, muscle biology remains largely underappreciated in the interpretation of vaccine responses and in the rational design of adjuvants.

At baseline, skeletal muscle contains relatively few immune cells, and many vaccine strategies aim to induce local inflammation to enhance antigen-presenting cell infiltration, antigen uptake, and downstream humoral and cellular immune responses. However, accumulating evidence indicates that distinct inflammatory programs within muscle can differentially shape immune polarization, as exemplified by the Th2-skewing responses observed with nano-emulsion adjuvants such as MF59 where necrosis has also been observed.

These observations underscore the need to determine whether engagement of muscle regenerative pathways actively contributes to Th2-biased immunity, and whether such pathways can be bypassed or strategically leveraged depending on the immunological requirements of the target pathogen, such as in parasitic infections.

Moreover, interrogating how the muscle immune environment contributes to vaccine-induced immunity may provide insight into whether factors that impair muscle health such as aging and obesity also contribute to reduced vaccine efficacy. The muscle immune environment is profoundly altered with age; it remains an open question whether age-associated declines in vaccine efficacy are partially driven by changes in muscle composition and function, including the development of a sarcopenic microenvironment.

Collectively, these considerations position skeletal muscle not merely as a passive site of vaccine delivery, but as an active immunological organ whose regenerative and inflammatory state may be harnessed to improve vaccine precision and efficacy across diverse populations and disease contexts. However, despite this potential, much remains unknown about the complex interplay occurring during muscle inflammation, damage, and regeneration and further research into the molecular mechanisms is crucial to better understand the impact of the muscle environment during vaccination.

Furthermore, this review paper highlights the importance of expanding our understanding of the muscle-immune interface for vaccine development, but this can also have implications for other fields. For example, some immunotherapies have shown to lead to certain adverse effects on muscle health (122), and further investigating the muscle immune landscape can allow the development of next-generation immunotherapies where the muscle is protected during treatment.
